# Ecto-5′-nucleotidase (CD73) attenuates inflammation after spinal cord injury by promoting macrophages/microglia M2 polarization in mice

**DOI:** 10.1186/s12974-018-1183-8

**Published:** 2018-05-22

**Authors:** Shun Xu, Wei Zhu, Minghao Shao, Fan Zhang, Ji Guo, Haocheng Xu, Jianyuan Jiang, Xiaosheng Ma, Xinlei Xia, Xiuling Zhi, Ping Zhou, Feizhou Lu

**Affiliations:** 1 Department of Orthopedics, Huashan Hospital, Fudan University, No.12, Wulumuqi middle Road, Jingan District, Shanghai, 200040 China; 2 Department of Physiology and Pathophysiology, School of Basic Medical Sciences, Fudan University, No.138, Yixueyuan Road, Shanghai, 200032 China; 3 The Fifth People’s Hospital of Shanghai, Fudan University, Shanghai, China

**Keywords:** SCI, CD73, Microglial polarization, Adenosine A_2B_ receptor, p38

## Abstract

**Background:**

Immune activation, specifically activation of macrophages and resident microglia, leading to inflammation is a key component in the progression of spinal cord injury (SCI). Macrophages/microglia exist in two states—the classically activated M1 phenotype that confers pro-inflammatory effects or the alternatively activated M2 phenotype that confers anti-inflammatory effects. Ecto-5′-nucleotidase (CD73) is an immunosuppressive molecule intricately involved in adaptive and innate immune responses and is able to dephosphorylate AMP to adenosine. However, it is not known if CD73 is able to modulate the macrophages/microglia transformation between the M1 and M2 phenotypes.

**Methods:**

We used gene-deficient mice to determine the role of CD73 in macrophages/microglia polarization post-SCI in vivo. We used small interference RNA (siRNA) or pcDNA3.1 to inhibit or overexpress CD73 in BV2 cells to verify anterior discovery in vitro. A combination of molecular and histological methods was used to detect the macrophages/microglia polarization and explore the mechanism both in vivo and in vitro.

**Results:**

We found that SCI induced the upregulation of CD73 expression. CD73 deficient mice were noted to demonstrate overwhelming immune responses, few anti-inflammatory phenotype macrophages/microglia, and had a poorer locomotor recovery in comparison to wild-type mice that were also inflicted with SCI. In vitro studies found that CD73 suppression inhibited the expression of characteristic microglial anti-inflammatory polarization markers in BV2 cells, while the converse was noted in CD73 overexpression. Subsequent experiments confirmed that CD73 promoted microglia alternative activation by stimulating p38 MAPK.

**Conclusion:**

We were able to conclude that CD73 imparts neuroprotective effects by mediating macrophages/microglia polarization. These findings allow for better understanding of the modulatory factors involved in triggering the change in macrophages/microglia phenotypes, therefore uncovering additional molecules and pathways that may be targeted in the innovation of novel SCI therapies.

**Electronic supplementary material:**

The online version of this article (10.1186/s12974-018-1183-8) contains supplementary material, which is available to authorized users.

## Background

Spinal cord injury (SCI) typically causes irreversible motor and sensory deficits that result in enormous socioeconomic burden [1, 2]. There are approximately 2.5 million people globally who live with SCI, and this figure is thought to grow by 130,000 annually [3]. No pharmacological therapy that can effectively treat this condition exists [4]. The pathogenesis of SCI starts with mechanically inflicted trauma that incites primary and secondary injury phases. The secondary injury phase in SCI has been proven to function as a significant therapeutic window, during which neuroprotective treatment may be implemented in efforts to bolster functional recovery after SCI [5, 6]. Existing evidence implicates inflammation in the pathogenesis of secondary injury after SCI [6–8].

Macrophages are vital mediators of inflammation in central nervous system (CNS) injury. Macrophages have been found to either be of peripheral myeloid origin that infiltrates the CNS in response to injury or may originate from the CNS itself from the resident microglial population [9]. Microglia develop from embryonic yolk sac cells that migrate to the spinal cord at 11.5 days post-fertilization. Macrophages/microglia are extremely adaptable cells and are able to transform to functionally different phenotypes in response to changes in their microenvironment [10]. They are thought to exist as two polarized forms that confer opposite effects on the prognosis of CNS disorders—the M1 phenotype has been shown to be pro-inflammatory and cytotoxic, while the M2 phenotype demonstrates pro-repair and anti-inflammatory functions [9].

Cells regulate their immune and inflammatory responses via the binding of extracellular adenosine to G-protein-coupled adenosine receptors found on the surface of effector cells [11]. This is a critical process, and increasing volumes of literature highlights the neuroprotective role of nucleoside adenosine in injured brain and spinal cord tissues [12]. This beneficial phenomenon is thought to be due to the involvement of adenosine in the activation of macrophages and microglia [13, 14].

Ecto-5′-nucleotidase (CD73) is a 70 kDa glycosylated protein that exists on the external plasma membrane layer and functions to hydrolyze extracellular AMP into adenosine and phosphate [15]. About 85–95% of murine AMP-hydrolyzing capabilities are mediated by CD73, which is a major murine cerebral 5′-nucleotidase [16]. By modulating extracellular adenosine levels, CD73 is thought to be intricately associated to immune- and inflammatory-related cerebral developmental diseases [17, 18]. However, its effect on secondary spinal cord injury remains unknown.

The current study hypothesized that altered CD73 expression could therefore affect macrophages/microglia polarization through adenosine. Our experiments were two-pronged and first aimed to characterize CD73 expression in spinal cord after SCI and secondly to further delineate its role in the macrophages/microglia polarization both in vitro and in vivo.

## Methods

### Animals

C57BL/6 CD73 knock out (KO) male mice were kindly gifted by Prof. Thompson, Oklahoma Medical Research Foundation, Oklahoma City, USA. The Shanghai SLAC Laboratory Animal Co., Ltd. (Shanghai, China) supplied the wild-type (WT) male C57BL/6 mice.

### Surgery

Each mouse was inflicted with spinal crush injury at the midthoracic region (T8–T9) with Dumont-type forceps with a 0.2 mm spacer, as described previously [19]. Firstly, T8-T9 vertebrae laminectomies were carried out with a pair of fine rongeurs, with precautions taken to preserve the dura. Lateral compression of the spinal cord was applied to achieve a depth of 0.2 mm for 20 s. Mice were reared separately post-surgery and administered twice-daily manual bladder expression. Control mice received sham surgery with laminectomy but without damage to the spinal cord.

### Quantitative real-time PCR (qPCR) analysis

Total RNA extraction was performed with TRIzol reagent (Invitrogen, San Diego, CA, USA) based on the protocol supplied by the manufacturer, and the SYBR Green reagent was used in qPCR mRNA quantification. The housekeeping gene used was GAPDH, and the comparative ΔΔCT method was used to calculate mRNA relative expression levels.

### Assessment of motor function

Overall locomotor function was recorded utilizing the Basso, Beattie, and Bresnahan (BBB) locomotor recovery scale. It is a 21-item scale that is based on observations in stepping, coordination, and hindlimb movement in the open-field test, with 0 indicating no spontaneous locomotor activity and 21 indicating normal coordinated gait with parallel paw placement. Four groups (WT+Sham, WT+SCI, KO+Sham, KO+SCI) were assessed on post-operative days 1, 3, 7, 14, 21, 28, 35, and 60. Each mouse was observed for 4 min in the open-field test by an assessor blinded to the treatments.

### Histological and immunohistochemical assessment

Sham and SCI mice (*n* = 4) were deeply anesthetized with 10% chloral hydras (3.5 ml/kg, i.p.) 3 days after surgery. 0.9% NaCl was then used to perfuse the mice, followed by 4% paraformaldehyde in 0.01 M phosphate-buffered saline (PBS, pH = 7.4). Spinal cord tissue at the region of injury were dissected with a 0.5 margin on each side of the lesion and embedded in paraffin. Hematoxylin-eosin (HE) staining was then used for histopathological assessment on 25-μm-thick transverse paraffin sections mounted on poly-L-lysine-coated slides. The sections were also subjected to Nissl staining by incubation in 1% cresyl violet acetate and were examined under a light microscope. For immunohistochemical analysis of TNF-α, IL-1β, and CD73, paraffin was first removed from the sections, endogenous peroxidase blocked by a 10-min H_2_O_2_ incubation and 10 min methanol incubation, and finally for 30 min in serum-blocking solution. Sections were then incubated with TNF-α (1:100, Abcam, ab6671), IL-1β (1:100, Abcam, ab9722), and CD73 (1:100, Abcam, ab175396) antibodies for 1 h, followed by incubation with HRP-conjugated anti-rabbit secondary antibodies for 30 min. DAB was then added to the sections and incubated for 10 min to allow visualization of brain segments containing bound antibodies. All incubation processes were carried out at room temperature. A Nikon ECLIPSE Ti microscope (Nikon, Japan) was used for imaging. Semi-quantification of integrated optical density (IOD) and area was done with the help of Image Pro Plus 6.0.

### Immunofluorescence assessment

As described previously, samples of spinal cord tissue were extracted at day 3 post-surgery. After transfection for 24 h and LPS/IL-4 induction for 8 h, BV2 cell samples were fixed with 4% paraformaldehyde in 0.1 M phosphate buffer (pH 7.4) for 15 min. All samples were then immersed for 1 h with 1% bovine serum albumin and 0.3% Triton X-100 in order to block all reactions. This was followed by an overnight incubation at 4 °C with the following primary antibodies: CD68 (1:100; Abcam, ab201845), CD73 (1:100, Abcam, ab175396), iNOS (1:100; Abcam, ab49999), and Arg1 (1:100, Abcam, ab133543). The next day, all samples underwent PBS washing and 2 h room temperature incubation with their corresponding secondary antibody: Dylight (Dy)488- and Dy594-conjugated secondary antibodies (all 1:1000; Jackson ImmunoResearch, West Grove, PA). Imaging was performed with either the Nikon ECLIPSE Ti microscope (Nikon, Japan) or Olympus FV 1000 confocal microscope (Olympus, Tokyo, Japan).

### Inflammatory cytokine array

The conditioned media containing transfected and IL-4 treated BV2 cells as well as lysate from injured spinal tissue were concentrated and analyzed with the Ray Biotech (Norcross, GA) Mouse Inflammation Antibody Array G series I and processed in accordance with the manufacturer’s protocol.

### Western blot analysis

A RIPA buffer (25 mM Tris•HCl pH 7.6, 150 mM NaCl, 1% NP-40, 1% sodium deoxycholate, 0.1% SDS) was used to extract total protein for subsequent concentration analysis with the BCA assay. SDS-polyacrylamide gel electrophoresis was used to dissolve protein samples, which were then transferred to nitrocellulose membranes. Subsequently, a 1-h incubation with 5% skim milk in TBST (25 mM Tris, 150 mM NaCl, 0.05% Tween-20, pH 7.5) was used to block reactions. Membranes were then incubated with antibodies (1:1000 dilutions for all antibodies) overnight at 4 °C. A HRP-conjugated secondary antibody was then added to the sections for a 1-h incubation at ambient temperature, and section colors were subsequently developed with ECL. A gel imaging system (UVP LLC, Upland, CA, USA) was used to image results which were then measured using Gel-Pro Analyzer software (Media Cybernetics, Rockville, MD, USA).

### Cell cultures

BV2 cells were grown in DMEM (Gibco, Carlsbad, CA, USA) supplemented with 10% FBS (Gibco, Carlsbad, CA, USA), 50 g/ml streptomycin (Invitrogen, Carlsbad, CA, USA), and 50 U/ml penicillin in a humidified atmosphere of 95% air and 5% CO_2_.

### Construction and transfection of siRNA and pcDNA3.1 plasmids

Plasmid construction was guided by previous reports [20, 21]. Briefly, three CD73 DNA sequences (CGTTGGATACACTTCCAAA, GGAGGACACTCCAACACAT, and CAACGTGGTTTCTACATAT) were selected for designing the siRNA target. Based on the U6 siRNA expression vector, pRNAT-U6.1/Neo vector (GenScript Corp., Piscataway, NJ, USA), three CD73 siRNA plasmids were built. In addition, the CD73 gene was extracted from the pBluescript SK(±) vector and cloned into the unique BamHI and KpnI pcDNA3.1 expression vector cloning sites. All constructs were confirmed by sequencing. The plasmid siRNA-CD73 and pcDNA3.1-CD73 were transfected into BV2 cells using Lipofectamine™ 2000 reagent (Invitrogen, Carlsbad CA, USA) following the protocol stipulated by the manufacturer.

### Statistical analysis

All results are expressed as mean ± standard deviation. Student’s unpaired *t* tests and two-way analysis of variance (ANOVA) followed by Dunnett’s test were used to analyze data. A *p* value of less than 0.05 was considered to be statistically significant. All statistical analyses were done with the SPSS 14.0 software.

## Results

### CD73 was upregulated after SCI

**Fig. 1:**
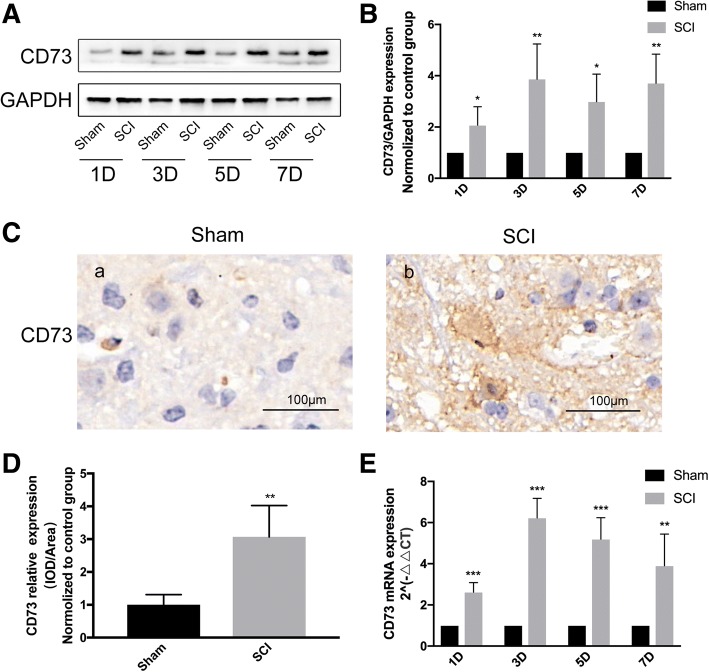
Ecto-5′-nucleotidase (CD73) expression was upregulated after SCI in mice. **a**–**b** Representative immunoblotting and quantification of CD73 at each time point after SCI or sham surgery. **c**–**d** Representative immunohistochemical staining for CD73 and quantification on the third day after SCI or sham surgery. **e** Relative CD73 mRNA expression assessed by real-time PCR at each time point after SCI or sham surgery. *p < 0.05, **p<0.01, ***p<0.001. Data are shown as the mean ± SD from four independent experiments

**Fig. 2:**
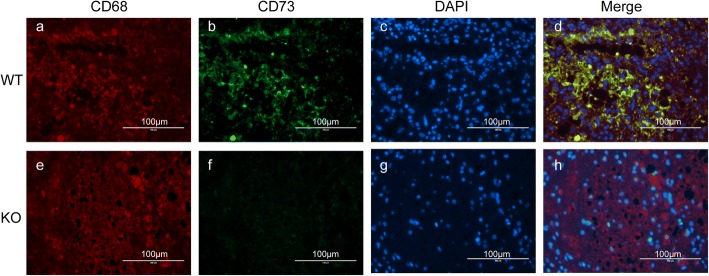
CD73 upregulation was mainly observed in microglia. Representative two-photon excitation images of immunofluorescence (IHF) of CD68 and CD73 acquired from WT mice (**a**-**d**) 3 days post-injury. CD73 KO mice (**e**-**h**) were used as control.

Existing evidence demonstrates that brain infarction results in significantly increased CD73 expression [17, 22]. However, the role of CD73 in SCI has not been reported. We utilized Western blot analysis and qPCR to quantify CD73 expression in response to SCI. Immunohistochemistry staining was also employed on the third day after SCI. Our data showed that both CD73 protein and mRNA expressions (Fig. 1) were significantly increased in mice who were subjected to SCI, indicating that CD73 may be related to the pathophysiological mechanisms of SCI. And the increased expression of CD73 3 days post-SCI was mainly observed in microglia and macrophages (Fig. 2).

### CD73 deficiency exacerbated motor dysfunction, inflammatory responses, and neuronal apoptosis induced by SCI

**Fig. 3:**
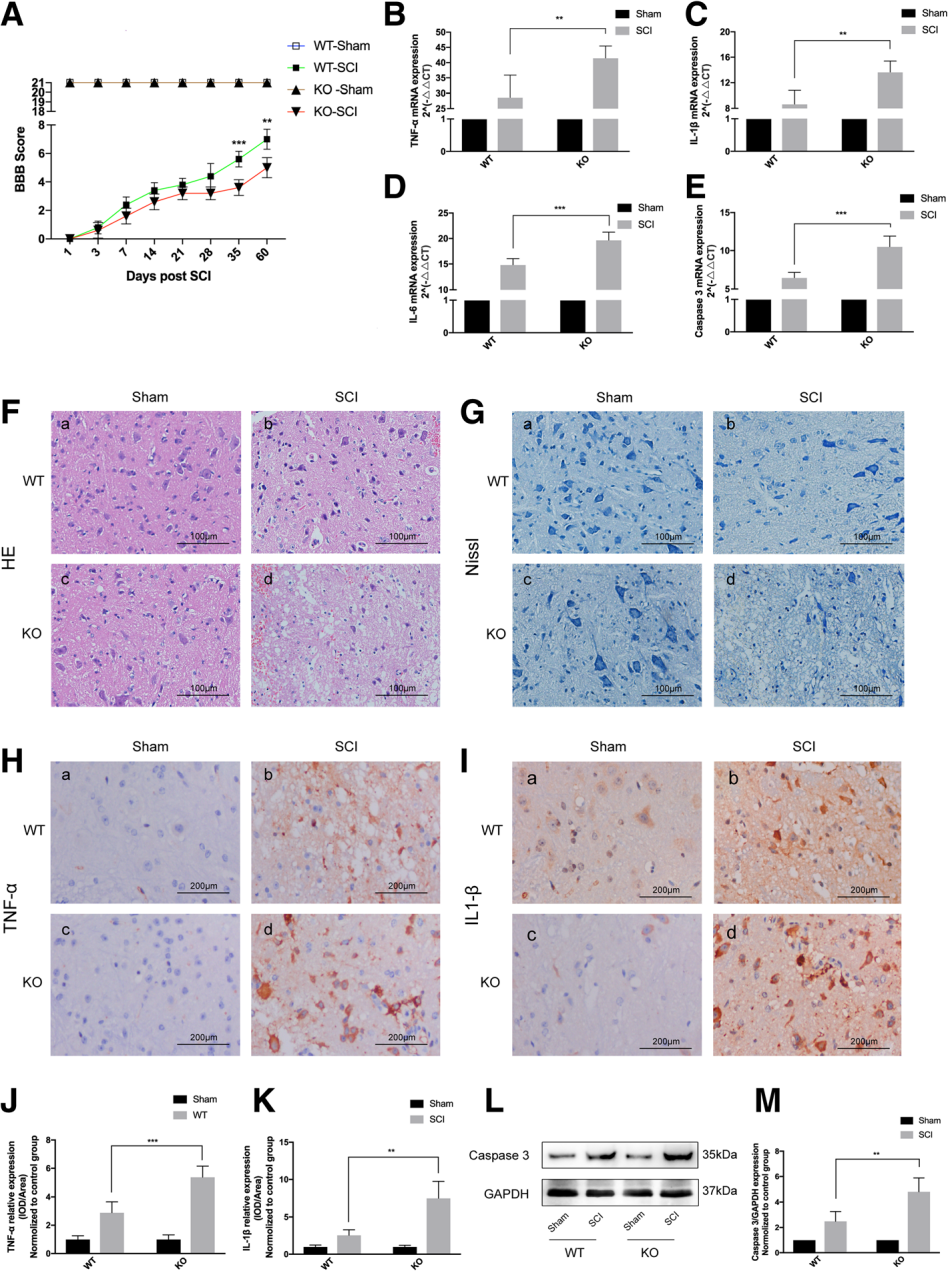
CD73 deficiency exacerbated the motor dysfunction, inflammatory responses, and neuronal apoptosis induced by SCI. **a** Degree of motor disturbance assessed by the Basso, Beattie, and Bresnahan (BBB) criteria at different time points after SCI or sham surgery of wild-type (WT) or CD73 knock out (KO) mice. **b**–**e** Relative mRNA expression of TNF-α, IL-1β, IL-6, and Caspase 3 on the third day after SCI or sham surgery in two groups. **f**, **g** Representative hematoxylin-eosin (HE) and Nissl staining of different groups 3 days after SCI or sham surgery. **h**–**k** Immunopositive particles for TNF-α and IL-1β and quantification 3 days post-injury or sham surgery in WT or CD73 KO mice. **l**, **m** Representative Western blotting and statistical comparison of Caspase 3 on the third day after SCI or sham surgery in WT or CD73-deficient mice. **p < 0.01, ***p < 0.001. Data are shown as the mean ± SD from four independent experiments

BBB locomotor recovery scale was utilized to evaluate motor dysfunction in both the CD73-knockout (KO) and wild-type (WT) mice that had been subjected to SCI. A BBB score of 0 was noted in the immediate period after SCI was inflicted. All groups of mice were then observed for the presence of spontaneous functional recovery for 60 days. The BBB scoring in WT mice was significantly superior to CD73-KO mice at the 35th day after injury (Fig. 3a). The CD73-KO group also expressed higher levels of TNF-α, IL-1β, IL-6, and Caspase 3 mRNA in comparison to the WT group at 3 days after SCI (Fig. 3b–e). Hematoxylin-eosin (HE) and Nissl staining were employed to evaluate histological features of myeloid tissue. Neuronal disorganization, swelling, and decrease in Nissl bodies were more obvious in the CD73-KO group at 3 days post-injury (Fig. 3f–g). Western blot and IHC were used to quantify TNF-α, IL-1β, and Caspase 3 protein expressions on the third day after SCI, and the results were similar to their mRNA expression (Fig. 3h–m).

### CD73 deficiency inhibited macrophages/microglia anti-inflammatory activation in vivo

Anti-inflammatory microglia phenotype is most effectively identified by the use of the arginase 1 (Arg1) marker. Arg1 acts to metabolize arginine into proline, polyamines, and ornithines that are crucial in matrix deposition and wound healing and matrix [23, 24]. Arg1 can effectively displace inducible nitric oxide synthase iNOS to downregulate nitric oxide production [25, 26]. Both Arg1 and iNOS are valuable markers that may be utilized to characterize anti-inflammatory activation and pro-inflammatory microglial phenotypes.

**Fig. 4:**
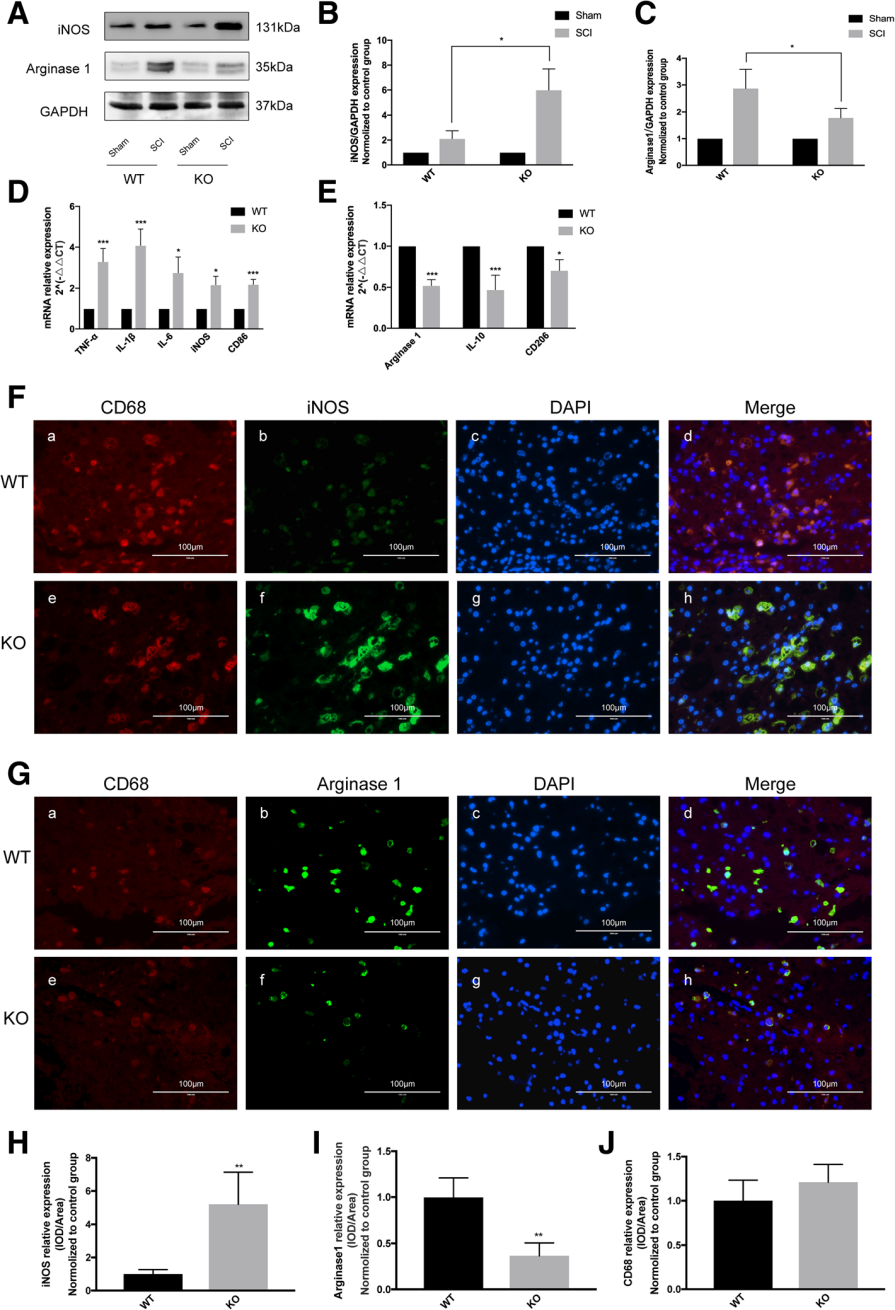
CD73 deficiency inhibited microglial anti-inflammatory activation in vivo. **a**–**c** Representative Western blotting of inducible nitric oxide synthase (iNOS) and arginase1 (Arg1) and quantification 3 days after SCI or sham surgery. **d**, **e** Microglial M1 polarization markers (TNF-α, IL-1β, IL-6, iNOS, CD86) and M2 polarization markers (Arg1, IL-10, CD206) mRNA expression identified by real-time PCR. **f**–**j** Representative two-photon excitation images of immunofluorescence (IHF) and statistical comparison of CD68 and iNOS/Arg1 acquired from WT or CD73 KO mice 3 days post-injury. *p < 0.05, **p < 0.01, ***p < 0.001. Data are shown as the mean ± SD from four independent experiments

On the third day post-SCI, CD73-KO mice had higher levels of iNOS compared to WT mice, while Arg1 expressions were decreased in CD73-KO mice in comparison to WT mice (Fig. 4a–c). At 3 days post-injury, mRNA expressions of pro-inflammatory activation markers were higher while mRNA expressions of anti-inflammatory activation markers were lower when compared to WT mice (Fig. 4d–e). Similar patterns of iNOS and Arg1 protein expressions were also revealed in IHF studies (Fig. 4f–j). CD68 was used as a marker of microglia and macrophages. These results imply that a CD73 deficiency may promote macrophages/microglia polarization to their pro-inflammatory phenotypes.

### CD73 deficiency inhibited microglial M2 polarization in vitro

**Fig. 5:**
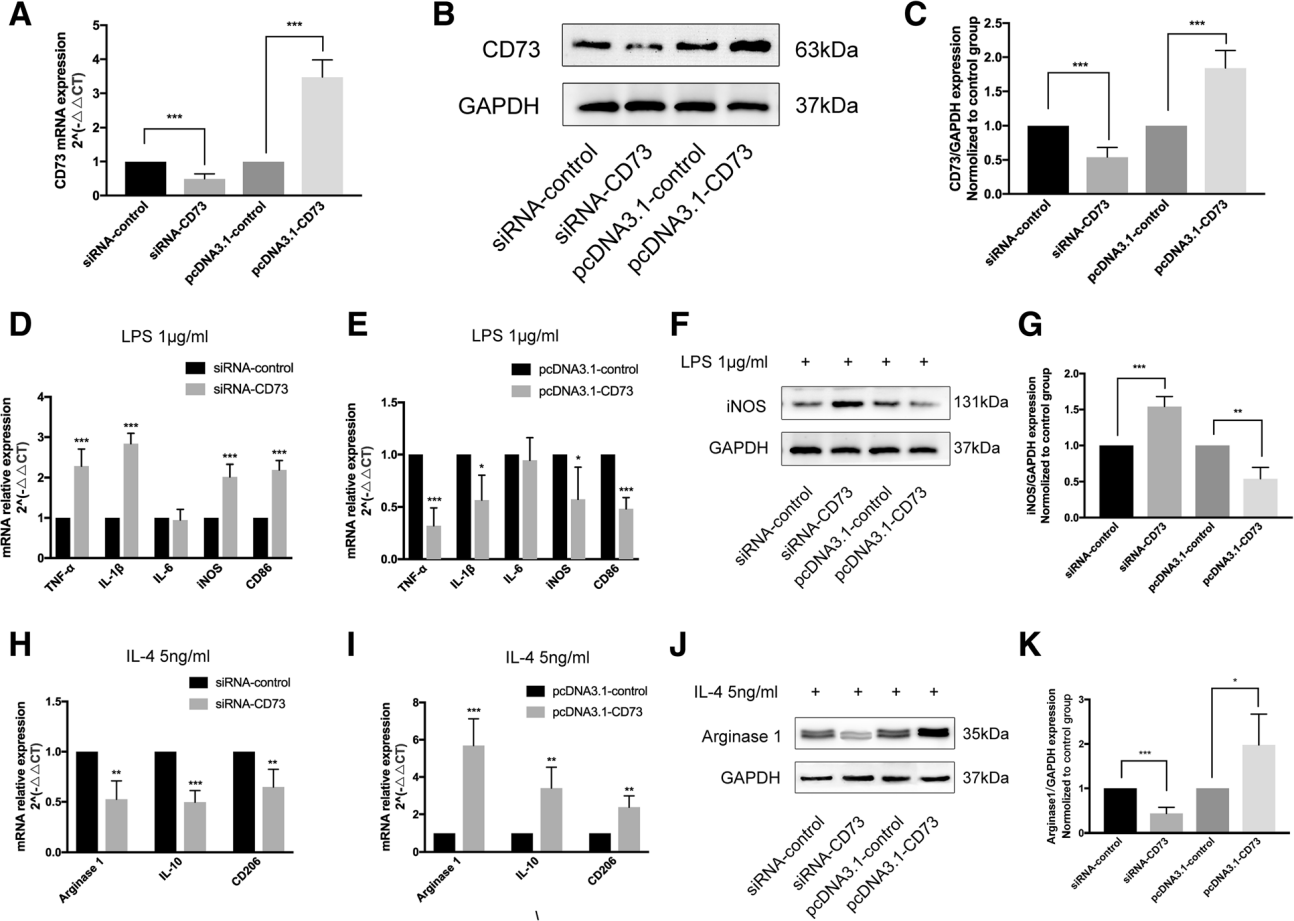
CD73 deficiency promoted microglial M1 polarization and inhibited microglial M2 polarization in BV2 cells, while CD73 overexpression had an opposite effect. After small interference RNA (siRNA) or pcDNA3.1 transfection for 24 h, BV2 cells were challenged with LPS or IL-4 for another 8 h to activate M1 or M2 activation. Then, Western blot and real-time PCR were applied to determine the expression of M1 or M2 markers in BV2 cells under different culture conditions. **a** Real-time PCR estimates the efficiency of CD73 interference and overexpression in BV2 cells 24 h after transfection. **b**, **c** Representative immunoblotting of CD73 expression and quantification 24 h after transfection. **d**, **e**, **h**, **i** After administration of LPS or IL-4, microglial M1 polarization markers (TNF-α, IL-1β, IL-6, iNOS, CD86) or M2 polarization markers (Arg1, IL-10, CD206) mRNA expression were identified by real-time PCR in CD73 downregulated or upregulated BV2 cells. **f**, **g**, **j**, **k** Representative Western blotting and quantification of iNOS or Arg1 expression induced by LPS/IL-4 in CD73 different expression BV2 cells. *p < 0.05, **p < 0.01, ***p < 0.001. Data are shown as the mean ± SD from four independent experiments

**Fig. 6:**
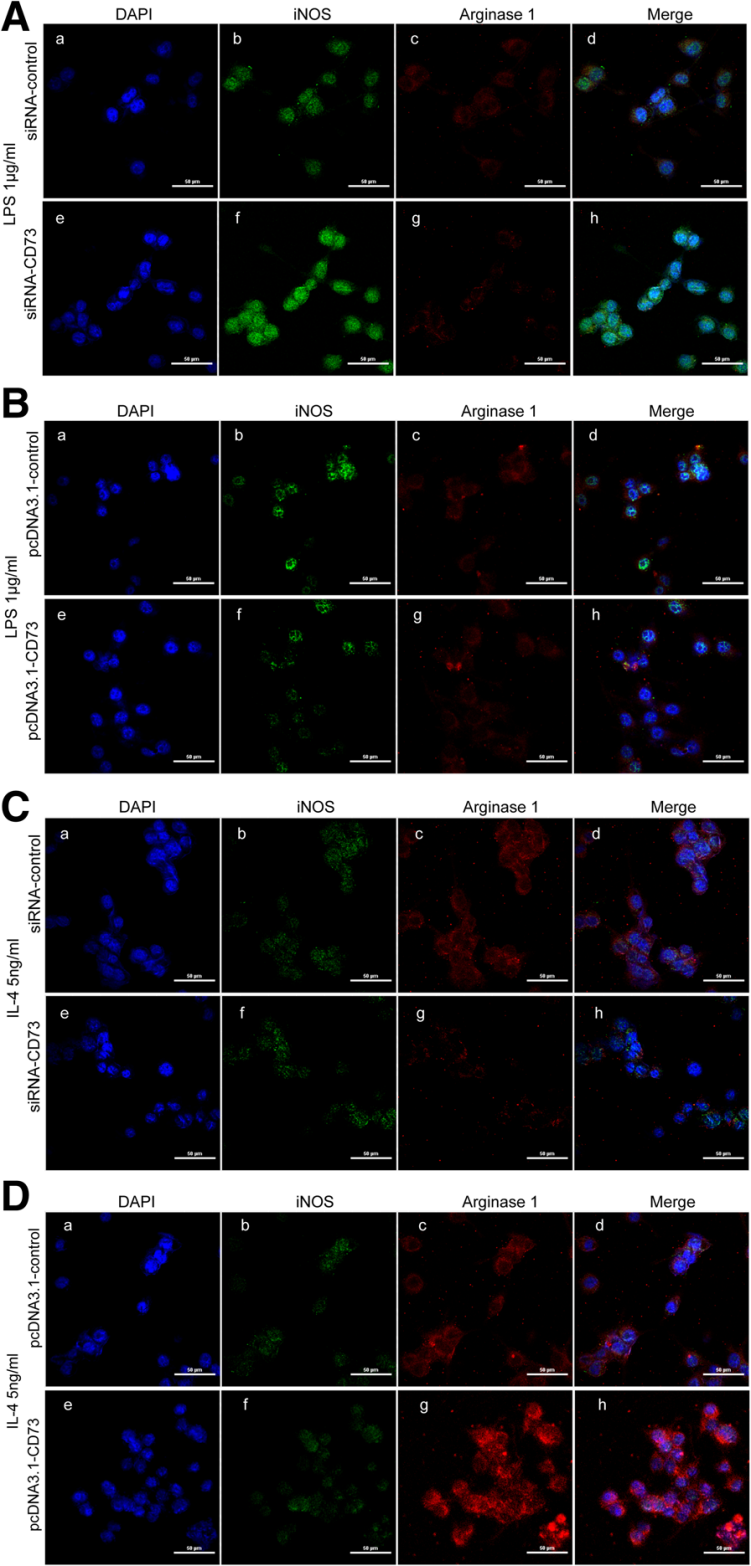
CD73 deficiency promoted microglial M1 polarization and inhibited microglial M2 polarization in BV2 cells, while CD73 overexpression had an opposite effect. After siRNA or pcDNA3.1 transfection for 24 h, BV2 cells were challenged with LPS or IL-4 for another 8 h to activate M1 or M2 activation. **a**–**d** Representative two-photon excitation images of IHF acquired using confocal microscope in siRNA-CD73 or pcDNA3.1-CD73 group BV2 cells activated by LPS or IL-4

We constructed BV2 cells that either had downregulated or upregulated CD73 expression in order to further discern the CD73 effect on microglial activation. Small interference RNA (siRNA) or pcDNA3.1 was successful in inhibiting or overexpressing CD73 at both the mRNA and protein levels in BV2 cells (Fig. 5a–c). In this study, we employed LPS or IL-4 to stimulate macrophages/microglia into M1 or M2 phenotype in cultured BV2 cells [27, 28]. In BV2 cells that had downregulated CD73, LPS stimulation significantly increased the expression of M1 mRNA markers (TNF-α, IL-1β, iNOS, CD86) compared with control BV2 cells (Fig. 5d). However, overexpression of CD73 was counterproductive (Fig. 5e). Our results also demonstrated that CD73 did not affect IL-6 expression in BV2 cells that were exposed to LPS. The protein expression of iNOS was similar to its mRNA expression in both groups (Fig. 5f, g). When treated with IL-4, the mRNA expression of M2 markers (IL-10, Arg1, CD206) was significantly reduced in the CD73 downregulated group compared with the control group (Fig. 5h). In contrast, CD73 overexpression leads to a significant increase in M2 markers (IL-10, Arg1, CD206) (Fig. 5i). Arg1 protein expression showed the same pattern as its mRNA expression (Fig. 5j, k). In addition, we applied IHF to explore the effect of CD73 expression on microglia polarization. It is observed that both the iNOS and Arg1 expression results concur with the Western blot and real-time PCR findings (Fig. 6).

### Differentially expressed proteins were screened via inflammatory cytokine assay

To determine the role of CD73 in microglia/microphages polarization, an inflammatory cytokine assay was used to quantify inflammatory cytokine expression in CD73 upregulated or downregulated BV2 cells at 8 h after being treated with IL-4. Tissues at the site of SCI in both CD73-KO mice and WT mice were also included in this analysis.

**Fig. 7:**
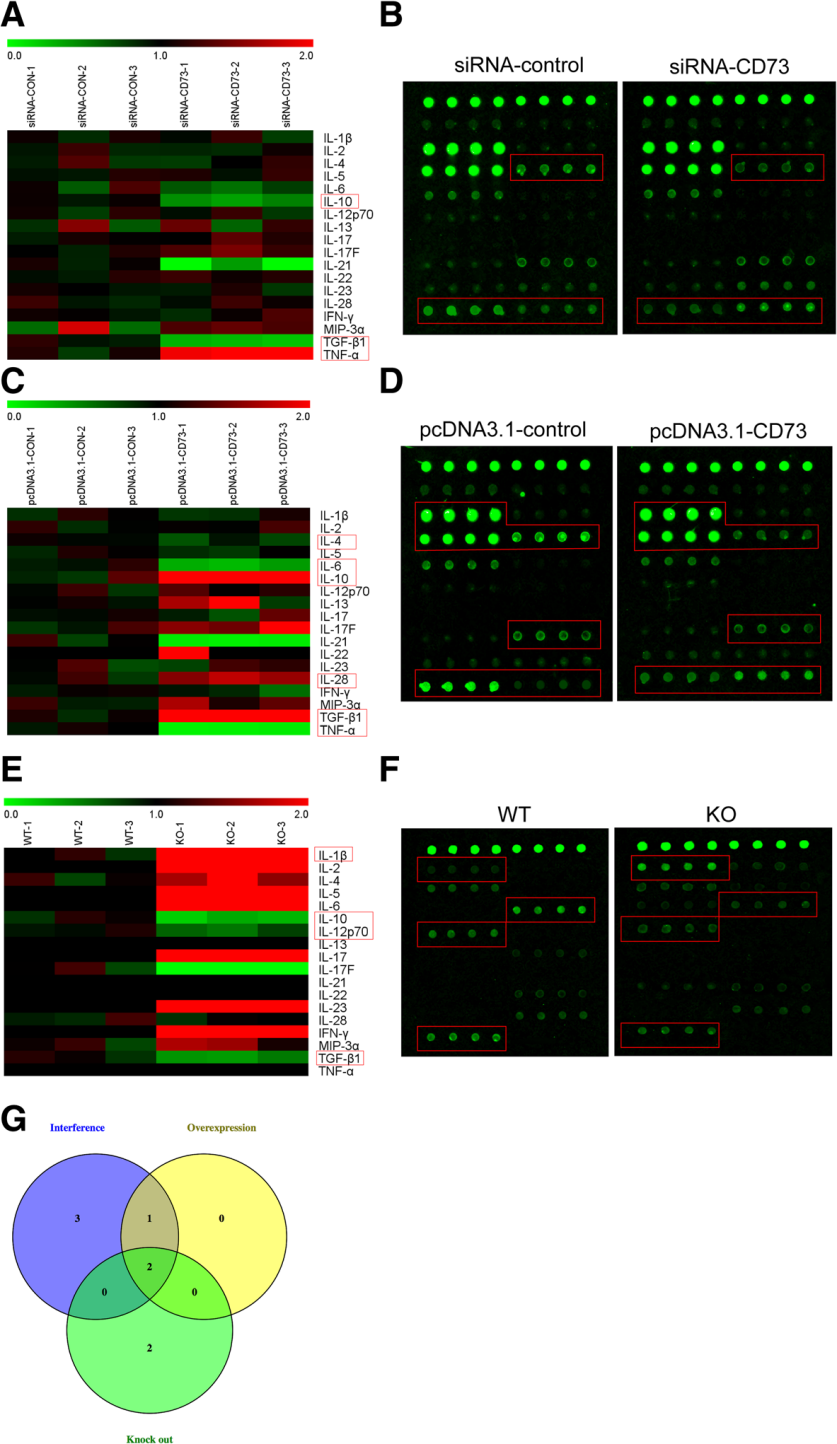
Differentially expressed proteins screened via inflammatory cytokine array. After siRNA or pcDNA3.1 transfection for 24 h, BV2 cells were challenged with IL-4 for another 8 h to activate M2 activation, then the content of inflammatory cytokines in the supernatant fluid of cultured BV2 cells was detected by inflammatory cytokine array. Besides, the inflammatory cytokines in the lesion areas after SCI both from CD73 KO mice and WT mice were included into this detection. The differentially expressed proteins screening criteria were as follows: fluorescence intensity > 1000, fold change > 1.5, and *p* < 0.05. **a**–**f** Heatmaps of inflammatory cytokines fold changes and representative photographs of inflammatory cytokine array reflected the protein expression in CD73 differentially expressed cells or tissues. The differentially expressed proteins were highlighted with red boxes. **g** Venn diagram depicting the overlap of differentially expressed proteins identified in three groups. IL-10 and TGF-β were the intersection

The differentially expressed proteins screening criteria were as follows: fluorescence intensity > 1000, fold change > 1.5, and *p* < 0.05. We found that TNF-α was increased in the siRNA-CD73 group, while IL-10 and TGF-β1 were reduced (Fig. 7a, b). In addition, the pcDNA3.1-CD73 group expressed higher IL-10 and TGF-β1 and lower IL-4, IL-6, IL-28, and TNF-α (Fig. 7c, d). We also found that IL-10 and TGF-β1 expressions were reduced, while IL-1β and IL-12p70 were highly expressed in CD73-deficient mice in comparison to WT mice at day 3 post-SCI (Fig. 7e–f). Both IL-10 and TGF-β1, which are closely related to M2 macrophages/microglia anti-neuroinflammatory function, were at the intersection of the three differentially expressed proteins groups, indicating that CD73 was involved in macrophages/microglia alternative activation.

### CD73 augments microglial M2 polarization via A_2B_ adenosine receptor activation

**Fig. 8:**
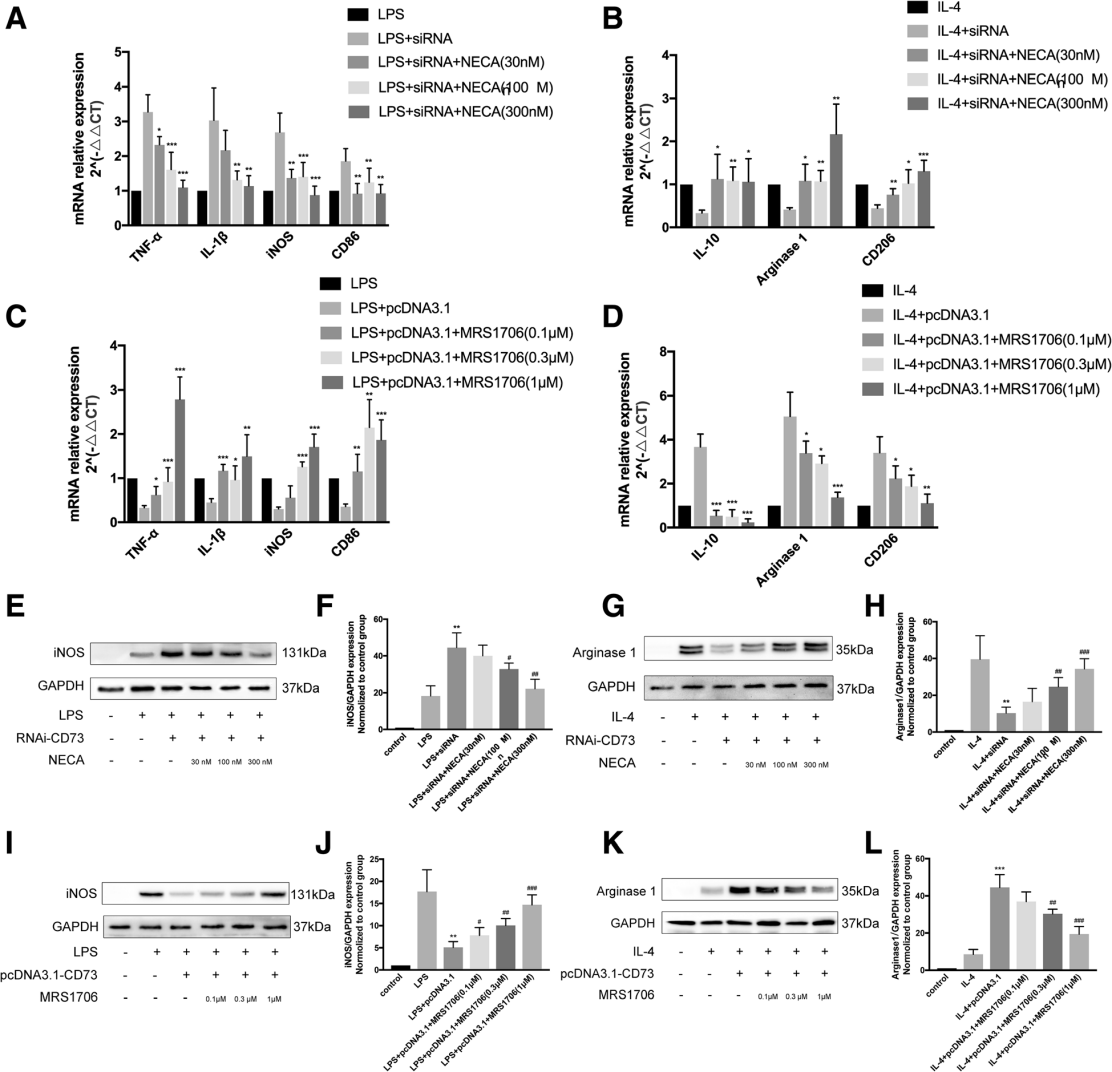
CD73 augments microglial M2 polarization via A_2B_ adenosine receptor activation. **a**, **b** The mRNA expression of microglial M1/M2 markers in BV2 cells challenged with LPS/IL-4 in presence or absence of siRNA or NECA. *p < 0.05, **p < 0.01, ***p < 0.001. **c**, **d** The mRNA expression of microglial M1/M2 markers in BV2 cells challenged with LPS/IL-4 in presence or absence of pcDNA3.1 or MRS1706. *p < 0.05, **p < 0.01, ***p < 0.001. **e**–**h** The iNOS/Arg1 protein expression in BV2 cells challenged with LPS/IL-4 in presence or absence of siRNA or NECA and statistical comparison. **p < 0.01 versus LPS/IL-4, ^#^*p* < 0.05, ^##^*p* < 0.01, ^###^*p* < 0.001 versus LPS/IL-4 + siRNA. **i**–**l** The iNOS/Arg1 protein expression in BV2 cells challenged with LPS/IL-4 in presence or absence of pcDNA3.1 or MRS1706 and statistical comparison. **p < 0.01, ***p < 0.001 versus LPS/IL-4, ^#^*p* < 0.05, ^##^*p* < 0.01, ^###^*p* < 0.001 versus LPS/IL-4 + pcDNA3.1. Data are shown as the mean ± SD from four independent experiments

To determine the subtype of adenosine receptor that mediates the CD73 ability to enhance alternative activation, we exposed LPS/IL-4 activated BV2 cells with an AR agonist and antagonist. We found NECA, a nonselective AR agonist, to be an inducer of M2 polarization in siRNA-CD73 BV2 cells. Not only did NECA decrease the mRNA expression of M1 markers (TNF-α, IL-1β, iNOS, CD86) treated with LPS, it also augmented M2 markers ((IL-10, Arg1, CD206) mRNA expression in cells treated with IL-4 (Fig. 8a–b). We also found that the selective A_2B_ adenosine receptor antagonist MRS1706 was able to reverse the effect of CD73 overexpression on microglia, which is reflected in the mRNA expression profiles of M1/M2 markers in cells that were treated with LPS/IL-4 (Fig. 8c–d). Protein expression levels of iNOS and Arg1 paralleled their mRNA expression (Fig. 8e–l).

### CD73 stimulates alternate microglial activation via p38

**Fig. 9:**
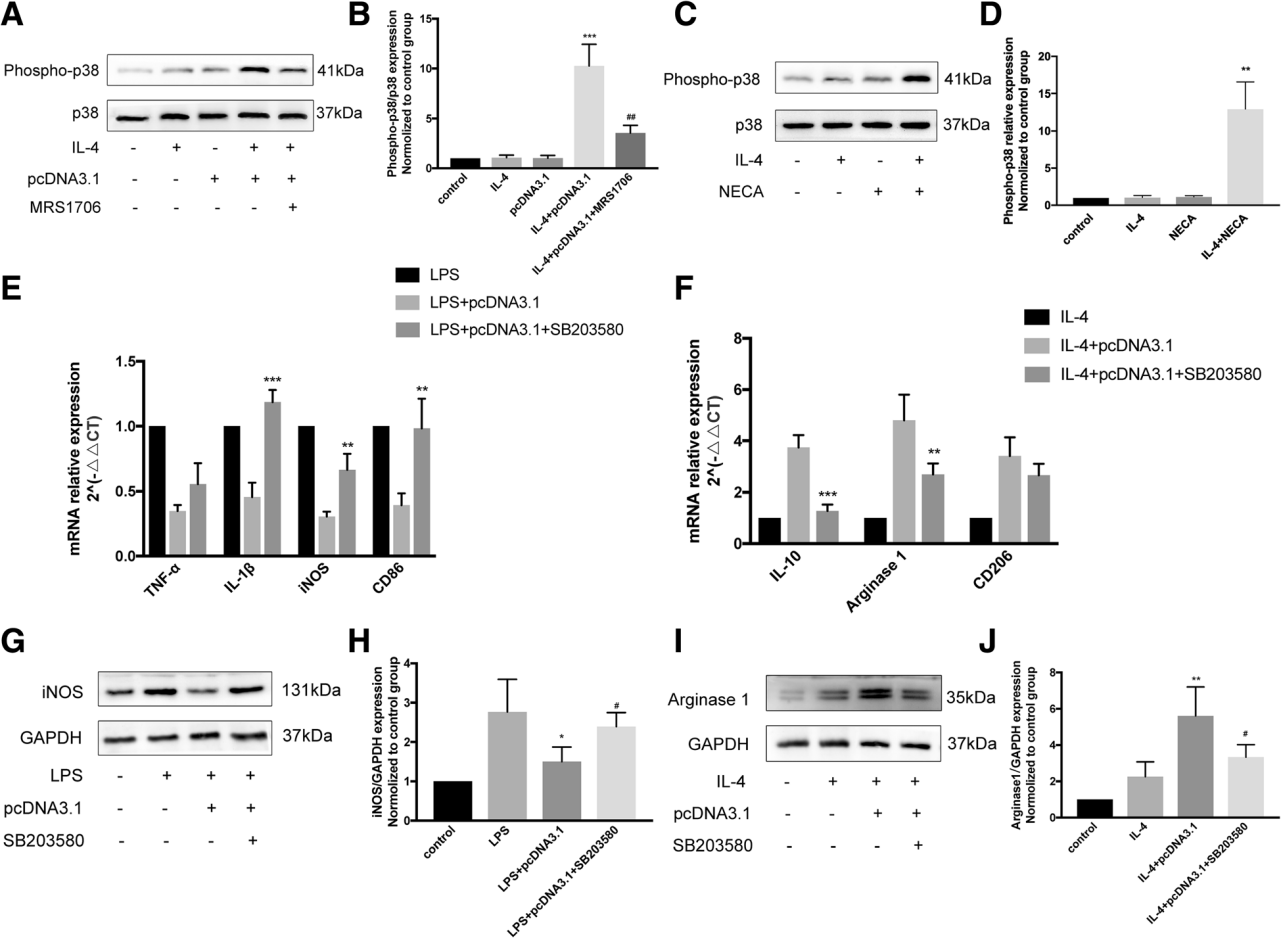
p38 activation was required for the stimulatory effect of CD73 on alternatively activated microglia. **a**, **b** Immunoblotting of phosphorylated form of p38 in BV2 cells challenged with IL-4 in presence or absence of pcRNA3.1 or MRS1706 and statistical comparison. ***p < 0.001 versus IL-4, ^##^*p* < 0.01 versus IL-4 + pcDNA3.1. **c**, **d** Immunoblotting of phosphorylated form of p38 in BV2 cells challenged with IL-4 in presence or absence of NECA and statistical comparison. **p < 0.01 versus IL-4. **e**, **f** The mRNA expression of microglial M1/M2 markers in BV2 cells challenged with IL-4 in presence or absence of pcDNA3.1 or SB203580. **p < 0.01, ***p < 0.001. **g**, **h**, **i**, **j** Immunoblotting of iNOS/Arg1 in BV2 cells challenged with LPS/IL-4 in presence or absence of pcDNA3.1 or SB203580 and statistical comparison. *p < 0.05, **p < 0.01 versus LPS/IL-4, ^#^*p* < 0.05 versus LPS/IL-4 + pcDNA3.1. Data are shown as the mean ± SD from four independent experiments

Previous studies have shown that adenosine stimulates alternate activation of macrophages and microglia in a p38-dependent pathway. To further elucidate the role of this kinase in M2-microglial polarization, we utilized Western blot to detect the active, phosphorylated form of p38. CD73 overexpression in combination with IL-4 upregulated p38 activation, while MRS1706 inhibited this effect (Fig. 9a, b). On the other hand, NECA also increased the activation of p38 when administered concurrently with IL-4 (Fig. 9c, d). Microglia exposure to SB203580, a selective p38 pathway inhibitor, 30 min before LPS/IL-4 exposure inhibited the CD73 attenuating effect on the expression of M1 markers (TNF-α, IL-1β, iNOS, CD86) while also inhibiting the CD73 promoting effect on M2 markers expression at both the mRNA and protein levels (Fig. 9e–j). Taken together, we conclude that the CD73 stimulatory effect on alternative microglial activation is mediated via the p38 pathway.

## Discussion

Trauma inflicted on the CNS, either by SCI or traumatic brain injury (TBI), often results in widespread inflammation, which leads to marked neuropathology and limited functional recovery [6, 29]. Researchers have confirmed the accumulation of pro-inflammation cytokines that encompasses TNFα, IL-1β, and IL-6 are able to activate microglia and astrocytes, resulting in a secondary cytotoxic response involving free-radical and vasoactive amines, eventually culminating in neuronal apoptosis [30, 31]. The current study revealed an upregulation of pro-inflammation cytokines in spinal cord tissue that peaked at 3 days post-SCI, and Caspase 3 mRNA expression indicates that significant neuronal apoptosis may be taking place, especially on the third day after SCI (Additional file 1). Based on these results, we can safely conclude that neuroinflammation plays a crucial role in the secondary injury phase of SCI.

CD73, a glycosylphosphatidylinositol (GPI) anchored cell surface protein, has a central role in adenosine signaling, working to catalyze AMP into phosphate and adenosine. This process has been linked to the proliferation, migration, invasion, and drug resistance of various cancer cells [20, 32, 33]. However, literature is scarce with regard to the effects of CD73 in SCI. Baud et al. reported that CD73 is abundant in neurons and gliocytes [34]. In 1997, Braun et al. described that focal cerebral ischemia enhanced glial expression of CD73 [22]. Additionally, Petrovic-Djergovic et al. demonstrated that CD73 was able to reduce infarcted area in an ischemic brain by regulating leukocyte trafficking [35]. In the bilateral common carotid artery stenosis model of cerebral hypoperfusion, Hou et al. demonstrated significantly elevated pro-inflammation cytokine levels in the presence of CD73 deficiency [17]. Together, these studies provide important insights into the neuroprotective effect of CD73 on the CNS. In the current study, SCI induced microglia CD73 expression (Figs. 1 and 2). CD73-deficient mice demonstrated worse motor dysfunction, more potent immune responses, as well as increased tissue destruction and cell apoptosis (Fig. 3). In the current study, no changes in BBB score were found at 3 days post-injury, the time point at which CD73 were upregulated. This discrepancy could be attributed to the plasticity of motor system after SCI. The recovery process after SCI can go on for several years and probably depends on the reorganization of circuits that have been spared by the lesion [36]. Within the first days after SCI, the initial recovery of function is mostly due to metabolic changes at the site of damage [37]. Synaptic plasticity in pre-existing pathways and the formation of new circuits through collateral sprouting of lesioned and unlesioned fibers occur subsequently [38]. So at 3 days post-injury, it seems possible that no changes in the BBB score between CD73 knocked out mice and wild-type mice because the synaptic plasticity and formation of new circuits have not even started. We considered that this also may be the reason why so many researches demonstrated different SCI therapies worked until 2 weeks after injury evaluated by BBB score [39–41]. In the research of Hsu et al., they demonstrated MMP-2 was neuroprotective after SCI, and the locomotor scores show a significant difference between MMP-2 deficient mice and wild-type mice until 42 days post-injury [42]. We also tried micro-MRI to evaluate the spinal trauma, and the T2 images showed that CD73-deficient mice had more extensive marrow edema and enhancement at 3 days post-injury (Additional file 2). These results are congruent with those obtained in earlier studies.

As a crucial CNS immune cell, macrophages/microglia are the first cells to be recruited in response to tissue injury or infection. They are able to manifest in two states with opposing functional characteristics, known as M1 (the classical pro-inflammatory macrophages/microglia) and M2 (the alternatively activated anti-inflammatory macrophages/microglia) [43–45]. Kigerl et al. observed that IL-4 polarized M2-conditioned media stimulated axonal proliferation in cultured neurons [46]. David et al. identified the association between alternative activation of microglia and improved neurological outcome after SCI in mice [47]. Moreover, Anhui et al. showed that inflammation after SCI was inhibited by programmed cell death-1 (PD-1) promoted M2 polarization [45]. When interpreted as a whole, these papers demonstrate that microglia/macrophages M2 activation can mitigate spinal cord damage associated with SCI.

Interestingly, our research also revealed that CD73 had the ability to disrupt macrophages/microglia polarization. The expression of pro-inflammatory activation markers in CD73-KO mice was significantly elevated while anti-inflammatory activation marker expression was suppressed at both the mRNA and protein levels, indicating the CD73 had a protective effect on secondary injury in SCI by promoting microglia/macrophages expressed Arg1, IL-10, and other anti-inflammation cytokines (Fig. 4). The in vivo results are also reflected in the in vitro models in this study. There is a marked accumulation of proteins characteristic of M1 microglia cells in CD73-KO BV2 cells that were treated with LPS, while the enhancement of M2 microglial markers was more significant when the CD73-KO BV2 cells were treated with IL-4 (Figs. 5 and 6). Moreover, the inflammatory cytokine array detected relatively low expressions of IL-10 and TGF-β in CD73-deficient cells and injured spinal cord tissue (Fig. 7). It is therefore likely that CD73 is intricately related to microglia/macrophages activation. Our findings are in contrast to those of Eichin et al., who found that CD73 is not a necessary factor for the polarization of M2 macrophages [48]. A possible explanation for this might be the differing experimental microenvironments resulting in different CD73 responses in microglia and macrophages.

Extracellular adenosine stimulates a myriad of protective cellular responses that has the ability to restore homeostasis [11]. There is experimental evidence that adenosine confers a disruptive quality to the pathogenic processes that are triggered by acute traumatic injuries, hinting towards the potential neuroprotective properties of adenosine receptor activation [49–52]. The effects of adenosine are triggered when it binds to and activates between one and four G-protein-coupled transmembrane adenosine receptors (ARs), designated A_1_, A_2A_, A_2B_, and A_3_ [53, 54]. Prior studies have observed that LPS-induced production of IL-12 and TNF-α by microglia is mitigated when adenosine is allowed to interact with A_2A_ and A_3_ [55, 56]. Csoka et al. found that adenosine promoted alternative macrophage activation [13], Koscso et al. discovered adenosine augmented microglial IL-10 production, and both studies indicated that adenosine’s protective effect was dependent on activation of the adenosine A_2B_ adenosine receptor [14]. Hence, it could conceivably be hypothesized that CD73 promotes microglia/macrophages M2 polarization by adenosine signaling. In this study, using nonselective agonist and A_2B_ selective antagonist, we demonstrated that the A_2B_ receptor is primarily responsible for stimulating CD73 to trigger microglia alternative activation (Fig. 8).

External stress signals are conveyed to cellular nuclei via mitogen-activated protein kinases (MAPKs). MAPK signaling can be stimulated by A_2A_ and A_2B_ receptors [57], while the p38-activating ability of adenosine has been demonstrated in macrophages and microglia [58–60]. Furthermore, the participation of p38 in the alternative activation of macrophages via A_2A_ and A_2B_ receptors also has been reported [13, 14]. These results provide support for the hypothesis that p38 has a crucial role in allowing CD73 to modulate microglial M2 polarization. We found that although IL-4 or CD73 overexpression alone had no effect on p38 phosphorylation, a combination of both increased p38 activation, and this effect can be mimicked or counteracted by NECA or MRS1706 (Fig. 9a–d). Additionally, p38 pathway inhibition negated the stimulating effects of CD73 on microglial M2 polarization induced by IL-4 exposure (Fig. 9e–j).

## Conclusions

In conclusion, we demonstrate for the first time that CD73 has an anti-inflammatory role in SCI, which is attributable to its inhibition of macrophages/microglia M1 polarization and facilitation of macrophages/microglia M2 polarization. Specifically, our results showed that CD73 promoted microglia alternative activation through p38 phosphorylation following A_2B_ receptor activation. We propose that CD73 may be utilized as a target molecule for the development of novel therapeutic methods for SCI.

## Additional files

Additional file 1: Expression of pro-inflammatory cytokines and Caspase 3 after spinal cord injury (SCI) in mice. (A–D) Real-time PCR reaction at each time point after SCI or sham surgery for pro-inflammatory cytokines and Caspase 3. (E–G) Immunohistochemical staining for TNF-α and IL-1β and semi-quantitative analysis on the third day post-injury or sham surgery. *p < 0.05, **p < 0.01, ***p < 0.001. Data are shown as the mean ± SD from four independent experiments. (TIFF 4019 kb)

Additional file 2: CD73 deficiency increased marrow edema and enhancement. Representative micro-MRI images of spinal cord at 3 days post-injury. (PDF 459 kb)

## Abbreviations

Arg 1: Arginase 1
ARs: Adenosine receptors
BBB: Basso, Beattie, Bresnahan
CD73: Ecto-5′-nucleotidase
CNS: Central nervous system
GPI: Glycosylphosphatidylinositol
HE: Hematoxylin-eosin
IHC: Immunohistochemistry
IOD: Integrated optical density
KO: Knock out
MAPKs: Mitogen-activated protein kinases
PD-1: Programmed cell death-1
SCI: Spinal cord injury
siRNA: Small interference RNA
TBI: Traumatic brain injury
WT: Wild-type
