# Supplementary figures and images for: Ecto-5′-nucleotidase (CD73) attenuates inflammation after spinal cord injury by promoting macrophages/microglia M2 polarization in mice
Source: J Neuroinflammation. 2018 May 22;15:155. doi: 10.1186/s12974-018-1183-8 (PMC5964922; doi:10.1186/s12974-018-1183-8)

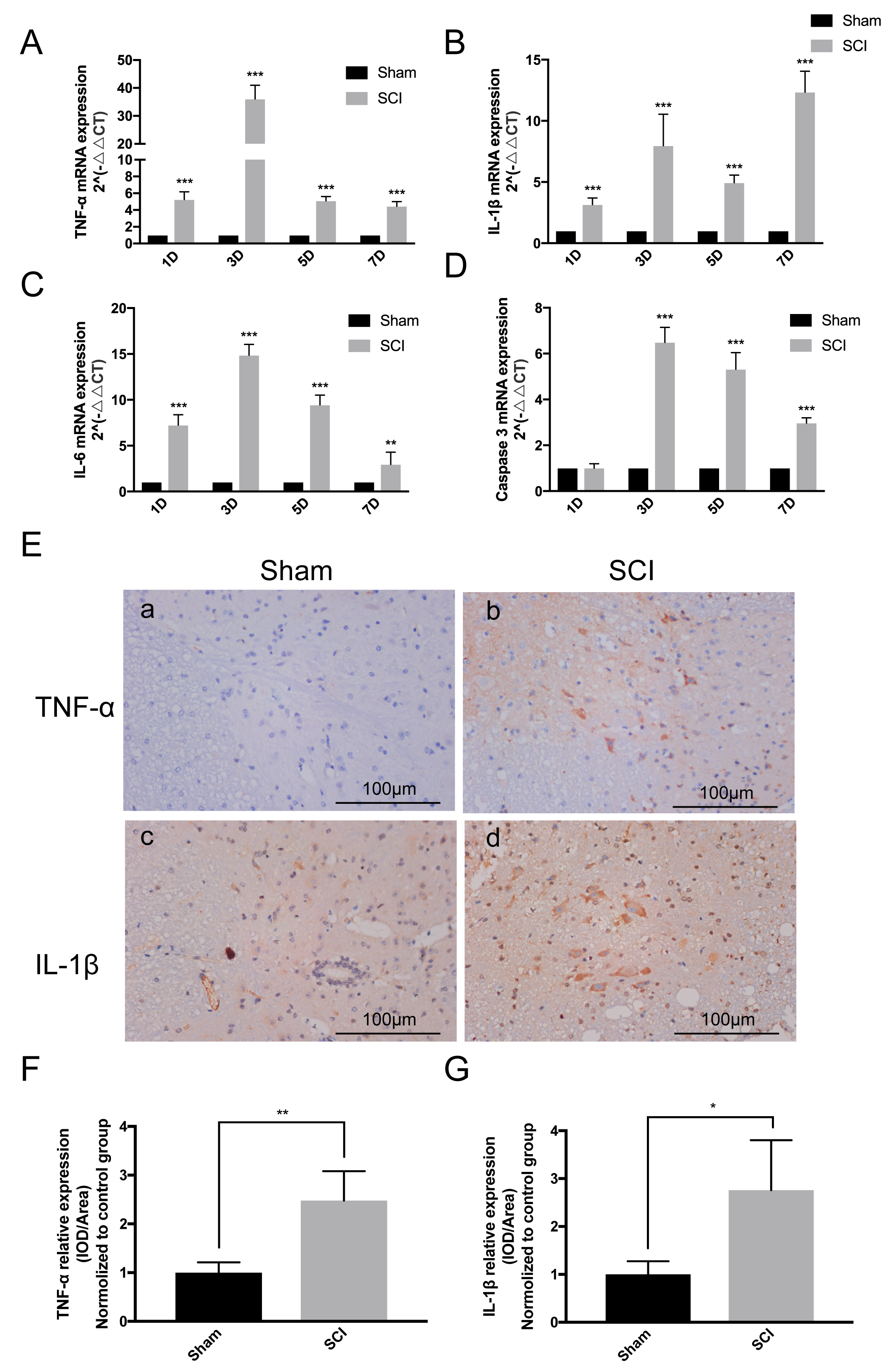

Supplement: Supplementary file 1 — Expression of pro-inflammatory cytokines and Caspase 3 after spinal cord injury (SCI) in mice. (A–D) Real-time PCR reaction at each time point after SCI or sham surgery for pro-inflammatory cytokines and Caspase 3. (E–G) Immunohistochemical staining for TNF-α and IL-1β and semi-quantitative analysis on the third day post-injury or sham surgery. *p < 0.05, **p < 0.01, ***p < 0.001. Data are shown as the mean ± SD from four independent experiments. (TIFF 4019 kb) [file 12974_2018_1183_MOESM1_ESM.tiff]

WT

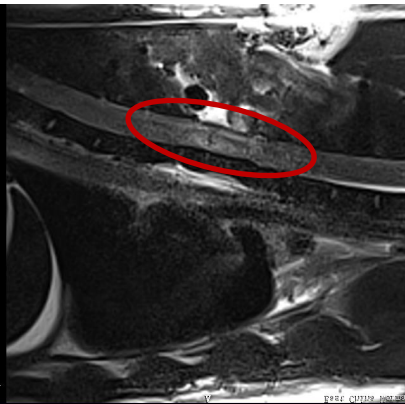

CD73 KO

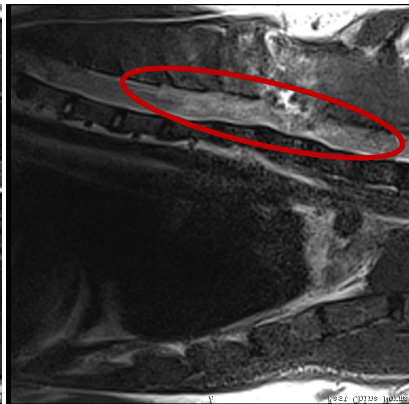

Supplement: Supplementary file 2 — CD73 deficiency increased marrow edema and enhancement. Representative micro-MRI images of spinal cord at 3 days post-injury. (PDF 459 kb) [file 12974_2018_1183_MOESM2_ESM.pdf]
